# Moderate-to-Severe Diarrhea and Stunting Among Children Younger Than 5 Years: Findings From the Vaccine Impact on Diarrhea in Africa (VIDA) Study

**DOI:** 10.1093/cid/ciac945

**Published:** 2023-04-19

**Authors:** Dilruba Nasrin, Yuanyuan Liang, Helen Powell, Ines Gonzalez Casanova, Samba O Sow, M Jahangir Hossain, Richard Omore, Doh Sanogo, Boubou Tamboura, Syed M A Zaman, Martin Antonio, Joquina Chiquita M Jones, Alex O Awuor, Irene N Kasumba, John B Ochieng, Henry Badji, Jennifer R Verani, Marc-Alain Widdowson, Anna Roose, Leslie P Jamka, Sharon M Tennant, Usha Ramakrishnan, Karen L Kotloff

**Affiliations:** Department of Medicine, Center for Vaccine Development and Global Health, University of Maryland School of Medicine, Baltimore, Maryland, USA; Department of Epidemiology and Public Health, University of Maryland School of Medicine, Baltimore, Maryland, USA; Department of Pediatrics, Center for Vaccine Development and Global Health, University of Maryland School of Medicine, Baltimore, Maryland, USA; Hubert Department of Global Health, Rollins School of Public Health, Emory University, Atlanta, Georgia, USA; Centre pour le Développement des Vaccins du Mali (CVD-Mali), Bamako, Mali; Medical Research Council Unit, The Gambia at the London School of Hygiene and Tropical Medicine, Banjul, The Gambia; Kenya Medical Research Institute, Center for Global Health Research, Kisumu, Kenya; Centre pour le Développement des Vaccins du Mali (CVD-Mali), Bamako, Mali; Centre pour le Développement des Vaccins du Mali (CVD-Mali), Bamako, Mali; Medical Research Council Unit, The Gambia at the London School of Hygiene and Tropical Medicine, Banjul, The Gambia; Medical Research Council Unit, The Gambia at the London School of Hygiene and Tropical Medicine, Banjul, The Gambia; Medical Research Council Unit, The Gambia at the London School of Hygiene and Tropical Medicine, Banjul, The Gambia; Kenya Medical Research Institute, Center for Global Health Research, Kisumu, Kenya; Department of Medicine, Center for Vaccine Development and Global Health, University of Maryland School of Medicine, Baltimore, Maryland, USA; Kenya Medical Research Institute, Center for Global Health Research, Kisumu, Kenya; Medical Research Council Unit, The Gambia at the London School of Hygiene and Tropical Medicine, Banjul, The Gambia; Division of Global Health Protection, US Centers for Disease Control and Prevention, Nairobi, Kenya; Division of Global Health Protection, US Centers for Disease Control and Prevention, Nairobi, Kenya; Department of Pediatrics, Center for Vaccine Development and Global Health, University of Maryland School of Medicine, Baltimore, Maryland, USA; Department of Medicine, Center for Vaccine Development and Global Health, University of Maryland School of Medicine, Baltimore, Maryland, USA; Department of Medicine, Center for Vaccine Development and Global Health, University of Maryland School of Medicine, Baltimore, Maryland, USA; Hubert Department of Global Health, Rollins School of Public Health, Emory University, Atlanta, Georgia, USA; Department of Medicine, Center for Vaccine Development and Global Health, University of Maryland School of Medicine, Baltimore, Maryland, USA; Department of Epidemiology and Public Health, University of Maryland School of Medicine, Baltimore, Maryland, USA; Department of Pediatrics, Center for Vaccine Development and Global Health, University of Maryland School of Medicine, Baltimore, Maryland, USA

**Keywords:** diarrhea, stunting, children, Africa, VIDA

## Abstract

**Background:**

Stunting affects >20% of children <5 years old worldwide and disproportionately impacts underserved communities. The Vaccine Impact on Diarrhea in Africa (VIDA) Study examined the association between an episode of moderate-to-severe diarrhea (MSD) and the risk of subsequent stunting in children <5 years living in 3 sub-Saharan African countries.

**Methods:**

In this prospective, matched, case-control study among children <5 years, data were collected over 36 months from 2 groups. “Children with MSD” visited a health center within 7 days of illness onset experiencing ≥3 loose stools/day plus sunken eyes, poor skin turgor, dysentery, intravenous rehydration, or hospitalization. “Children without MSD” were enrolled from the community within 14 days of the index MSD child; they were diarrhea-free during the previous 7 days and were matched to the index case by age, sex, and residence. Using generalized linear mixed-effects models, we estimated the effect of an MSD episode on odds of being stunted, defined as height-for-age *z*-scores <−2, at a follow-up visit 2–3 months post-enrollment.

**Results:**

The proportion of stunting at enrollment was similar when 4603 children with MSD and 5976 children without MSD were compared (21.8% vs 21.3%; *P* = .504). Among children not stunted at enrollment, those with MSD had 30% higher odds of being stunted at follow-up than children without MSD after controlling for age, sex, study site, and socioeconomic status (adjusted OR: 1.30; 95% CI: 1.05–1.62: *P* = .018).

**Conclusions:**

Children <5 years in sub-Saharan Africa without stunting experienced an increased likelihood of stunting during 2–3 months following an episode of MSD. Strategies for control of early childhood diarrhea should be integrated into programs intended to reduce childhood stunting.

## Teaser

Stunting disproportionately impacts young children living in low-income countries. In the VIDA study among children <5 years old living in sub-Saharan Africa, an episode of moderate-to-severe diarrhea conferred a 30% higher odds of becoming stunted during 60 days of follow-up.

An estimated 144 million children younger than 5 years are stunted globally, with over half in Africa and Asia [1]. Although the global prevalence of stunting has declined from 32.4% in 2000 to 21.3% in 2019, the number of stunted children in Africa increased from 49.7 million in 2000 to 57.5 million in 2019 due to a slower rate of decline in stunting and an increase in population growth [1, 2]. Stunting is associated with increased morbidity and mortality, as well as impaired cognitive development, educational performance, and economic productivity later in life [3–6]. Some studies suggest that the impact of stunting on development may continue into the next generation [7, 8]. Thus, it is vital to understand and prevent factors that contribute to stunting in order to reach the World Health Organization (WHO) goal of reducing stunting by 40% by 2025 [9].

Diarrheal disease accounts for an estimated 13.5% of stunting globally [3]. A pooled analysis, using data from over 20 years in low- and middle-income countries (LMICs), reported that the odds of stunting at 24 months of age increased by 16% with every 5% increase in the duration of total diarrheal episodes [10]. The Global Enteric Multicenter Study (GEMS), conducted from 2007 to 2011 in 7 Asian and African sites, reported that children aged 0–59 months with moderate-to-severe diarrhea (MSD) experienced more linear growth faltering during the 2–3 months following their illness than their matched controls [11].

The recently introduced rotavirus vaccine represents a major advance in the prevention of diarrheal morbidity and mortality in both developed and developing countries [12, 13]; however, whether the introduction of the rotavirus vaccine altered the association between MSD and subsequent linear growth has not been clearly elucidated. The Vaccine Impact on Diarrhea in Africa (VIDA) Study was a case-control study of the incidence, etiology, and clinical consequences of MSD in settings where the rotavirus vaccine had been introduced. VIDA used comparable methods to GEMS in the 3 GEMS sites in sub-Saharan Africa that introduced rotavirus vaccine into routine infant immunization programs from 2013 to 2014. It was expected to see a change in diarrheal etiology following the introduction of rotavirus vaccine and changes in other socioeconomic factors that contribute to the risk of stunting over time. This article presents updated data from African countries to evaluate the impact of an episode of MSD on the risk of moderate and severe stunting in children younger than 5 years of age, approximately 60 days after the episode began.

## METHODS

### Study Design

The VIDA study was a prospective, age-stratified, population-based, matched case-control study that was initiated following rotavirus vaccine introduction at 3 sites in sub-Saharan Africa: Basse and Bansang, The Gambia; Bamako, Mali; and Siaya County, Kenya. Each site maintained a censused population with an ongoing Demographic Surveillance System (DSS) from which cases and controls were enrolled. Details of the study design have been described previously [14, 15], with a brief overview here.

Participants from 3 age strata (0–11, 12–23, and 24–59 months) seeking care at sentinel health centers serving the DSS population were enrolled over 36 months between May 2015 and July 2018. MSD cases were defined as new and acute diarrhea (≥3 abnormally loose stools within the past 24 hours that started within the previous 7 days following at least 7 diarrhea-free days, based on caretaker's memory recall) in a child aged 0–59 months with at least 1 of the following criteria for MSD: dehydration based on the study clinician's assessment (sunken eyes, decreased skin turgor, or intravenous rehydration administered or prescribed), dysentery (visible blood in stools reported by the mother or observed by the study team), or hospitalization. The study team also enrolled 1–3 controls (without diarrhea for the previous 7 days) per case from the community within 14 days of the index case and matched by age (±2 months for <12 months, ±4 months for ≥12 months), sex, and residence. The study team visited each enrolled child at home approximately 2–3 months (50–90 days) after enrollment to measure growth and assess vital status.

### Data Collection

At enrollment, caretakers provided demographic, sociodemographic, epidemiological, and clinical information during a standardized interview. Caretakers received a simple memory aid card to record whether the child had diarrhea (≥3 abnormally loose stools/day) for 14 days following enrollment.

Trained staff measured length/height at enrollment and follow-up. Age was ascertained from source documents, including birth certificates and vaccination cards. Standing height was measured for children aged 2 years and older, whereas the length of children younger than 2 years was measured lying down using a Shorr board (Weigh and Measure, LLC). Length/height of each child was measured 3 times to the nearest 0.1 cm [16]. Measurements were repeated if any of the 3 measurements were more than 0.5 cm apart from the others. At follow-up, study staff collected children's clinical data from caretakers, including healthcare facility–diagnosed pneumonia, malaria, and diarrhea between enrollment and follow-up and measured the children again using the same techniques.

Before study initiation, staff received 3 days of anthropometry training, which included measuring length/height [16], followed by an exercise to assess intra- and interrater variability. Staff were considered trained when measurement error was within protocol limitations (0.5 cm). Local teams trained new staff and refreshed existing staff every 4–6 months. The core team (D. N., K. L. K.) visited each site approximately twice annually to train, observe, and review results.

### Ethics Review

This study was approved by the ethical review committees at the University of Maryland, Baltimore (HP-00062472); the Centers for Disease Control and Prevention (CDC) (reliance agreement 6729); The Gambia Government/Medical Research Council/Gambia at the London School of Hygiene and Tropical Medicine (1409); the Comité d’Ethique de la Faculté de Médecine, de Pharmacie, et d’Odonto-Stomatologie, Bamako, Mali (no number); and the Kenya Medical Research Institute Scientific and Ethics Review Unit in Siaya County, Kenya (SSE 2996).

### Statistical Analysis

#### Variable Definitions

Age was measured on a continuous scale and data analyzed using the study strata (0–11, 12–23, and 24–59 months). *z*-Scores, or standard deviation scores, were used to compare a child's growth with WHO global standards. The length/height-for-age *z*-score (HAZ) was calculated at enrollment and follow-up using the median of the 3 length/height measurements and age [17, 18]. Because of high malnutrition prevalence in the study sites, we used modified WHO 2006 cleaning criteria (HAZ < −6 or >6 with a change in HAZ >3 within the follow-up period) to exclude implausible values and to retain the values that are plausible in this population. Our primary outcome was a binary indicator of stunting using the standard WHO definition of HAZ < −2.0 [18]. The main predictor of interest was whether or not the child had MSD (case-control status) at the time of enrollment.

Demographic (age, sex, and study site), socioeconomic, and clinical (MSD status at enrollment, stunting status at enrollment, pneumonia, duration of diarrhea, and days to follow-up) characteristics were considered potential confounders and 4 interaction terms were considered: MSD status at enrollment × stunting status at enrollment, age group × stunting status at enrollment, study site × stunting status at enrollment, and sex × stunting status at enrollment. To capture socioeconomic status (SES), we defined indicator variables as shown in Table 1. We created an estimated continuous score for household possession of electricity, television, and refrigerator, called the “ETR score,” using principal components analysis (PCA) [19]. Both water and sanitation were categorized based on source or facility used as improved or unimproved using predefined criteria [20]. Additional clinical characteristics that were considered in the analysis include healthcare facility–diagnosed pneumonia between enrollment and follow-up by caretaker report, as diarrhea frequently precedes pneumonia in undernourished children [21], and a continuous measure of the number of days of diarrhea the child experienced beginning with the 7 days before enrollment until the 14 days following enrollment as reported in the memory aid. If a child was diarrhea-free at enrollment but developed diarrhea within 14 days following enrollment, this was captured by this variable. Seven or more diarrhea-free days indicated the episode ended, and no additional days were counted.

**Table 1.** Comparison of Demographic, Socioeconomic, and Clinical Characteristics of Children With Moderate-to-Severe Diarrhea (MSD) to Children Without MSD During May 2015 to July 2018 in 3 Sites in sub-Saharan Africa: Basse and Bansang, The Gambia; Bamako, Mali; and Siaya County, Kenya

	Children With MSD (n = 4603)	Children Without MSD (n = 5976)	*P*
**Demographic features**			
ȃAge in months, n (%)			
ȃȃ0–11	1629 (35.4)	2035 (34.1)	.031
ȃȃ12–23	1618 (35.2)	2038 (34.1)	
ȃȃ24–59	1356 (29.5)	1903 (31.8)	
ȃSite, n (%)			
ȃȃThe Gambia	1579 (34.3)	2046 (34.2)	.076
ȃȃMali	1542 (33.5)	1896 (31.7)	
ȃȃKenya	1482 (32.2)	2034 (34.1)	
Females, n (%)	2136 (46.4)	2767 (46.3)	.92
More than 2 under-5 children per caretaker, n (%)	2298 (49.9)	2778 (46.5)	<.001*
Crowding (>3 people sleeping per room), n (%)	1862 (40.5)	2405 (40.2)	.83
**Socioeconomic features**			
Caretaker completed ≥ primary school, n (%)	1549 (33.7)	2107 (35.3)	.09
Household possessions, n (%)			
Car	556 (12.1)	678 (11.4)	.25
Scooter	2362 (51.4)	2932 (49.1)	.02*
Bike	2437 (53.0)	3257 (54.6)	.109
Cart	1314 (28.6)	1609 (27.0)	.07
Phone	4409 (95.9)	5767 (96.6)	.05
Radio	3537 (76.9)	4658 (78.0)	.172
Agricultural land	2621 (57.0)	3590 (60.1)	.001*
Electricity	2446 (53.2)	3259 (54.6)	.15
Television	2148 (46.7)	2691 (45.1)	.10
Refrigerator	742 (16.1)	933 (15.6)	.48
Principal component, mean (SD)	00541 (1.45)	−0.00417 (1.44)	.74
Finished floor (missing = 20)	3337 (72.6)	4341 (72.8)	.78
Clean cooking fuel^a^ (missing = 18)	261 (5.7)	331 (5.5)	.78
Improved drinking water (missing = 1)	3871 (84.1)	5207 (87.2)	<.0001*
Improved sanitation	2891 (62.8)	3382 (56.6)	<.0001*
**Clinical variables**			
Stunted at enrollment, n (%)	1003 (21.8)	1270 (21.3)	.50
Diagnosed pneumonia,^b^ n (%)	79 (1.7)	76 (1.3)	.07
Days to follow-up, mean (SD)	66.8 (7.1)	66.1 (6.8)	<.0001*
Duration of diarrhea,^c^ median (range), days	5 (1-20)	0 (0-14)	<.0001*

Abbreviations: MSD, moderate-to-severe diarrhea; SD, standard deviation.

Clean fuel includes electric, propane, butane, or natural gas.

Subsequent morbidity information came from a questionnaire at ∼60 days follow-up.

Wilcoxon rank-sum test; 27.2% of children without MSD went on to develop diarrhea (≥3 abnormally loose stools in 1 day) during the 14 days after enrollment, as recorded by caretakers on the memory aid card.

*Significant, *P* < .05.

#### Analysis

We used data from VIDA, a matched case-control study. However, as the outcome of interest in this paper is stunting approximately 60 days following an episode of MSD, the MSD status at enrollment is an exposure in the analysis. Thus, to avoid confusion, we renamed cases as “children with MSD” and controls as “children without MSD.”

All children with height/length measured at both enrollment and follow-up were included in the analysis. Children who had missed height measurement at enrollment or at follow-up, who were lost to follow-up, or who died in the interim period were excluded.

To examine potential confounders between MSD and stunting at follow-up, children with MSD (cases at enrollment) were compared with children without MSD (controls at enrollment) for various demographic, socioeconomic, and clinical characteristics using chi-square tests for binary and categorical variables and *t* tests for continuous variables.

A chi-square test was used to compare the proportion stunted, separately at enrollment and follow-up, between children with MSD and children without MSD. McNemar's test was used to compare the proportions stunted at enrollment and follow-up, within each MSD status group (MSD vs without MSD). These tests also accounted for correlations between matched cases and controls introduced by the VIDA study design.

To determine the impact of an episode of MSD on growth during the follow-up period, we used a generalized linear mixed-effects model (GLMM) to estimate the odds of stunting at the follow-up visit, using a random effect to capture the non-independent observations introduced by the case-control matching in the original study design. In addition to MSD status at enrollment, stunting status at enrollment, duration to follow-up in days, and demographic, socioeconomic, and clinical characteristics were also included. We also included 4 interaction terms (MSD status at enrollment × stunting status at enrollment, age group × stunting status at enrollment, study site × stunting status at enrollment, and sex × stunting status at enrollment) in the GLMM.

Statistical Analysis System (SAS) version 9.4 (SAS Institute) was used for all summary statistics and associated tests, STATA/SE version 16 (StataCorp) was used to fit the GLMM, and figures were created using R version 3.6.1 (R Foundation for Statistical Computing). Unless otherwise stated, a *P* value less than .05 was considered statistically significant.

## RESULTS

**Figure 1.**
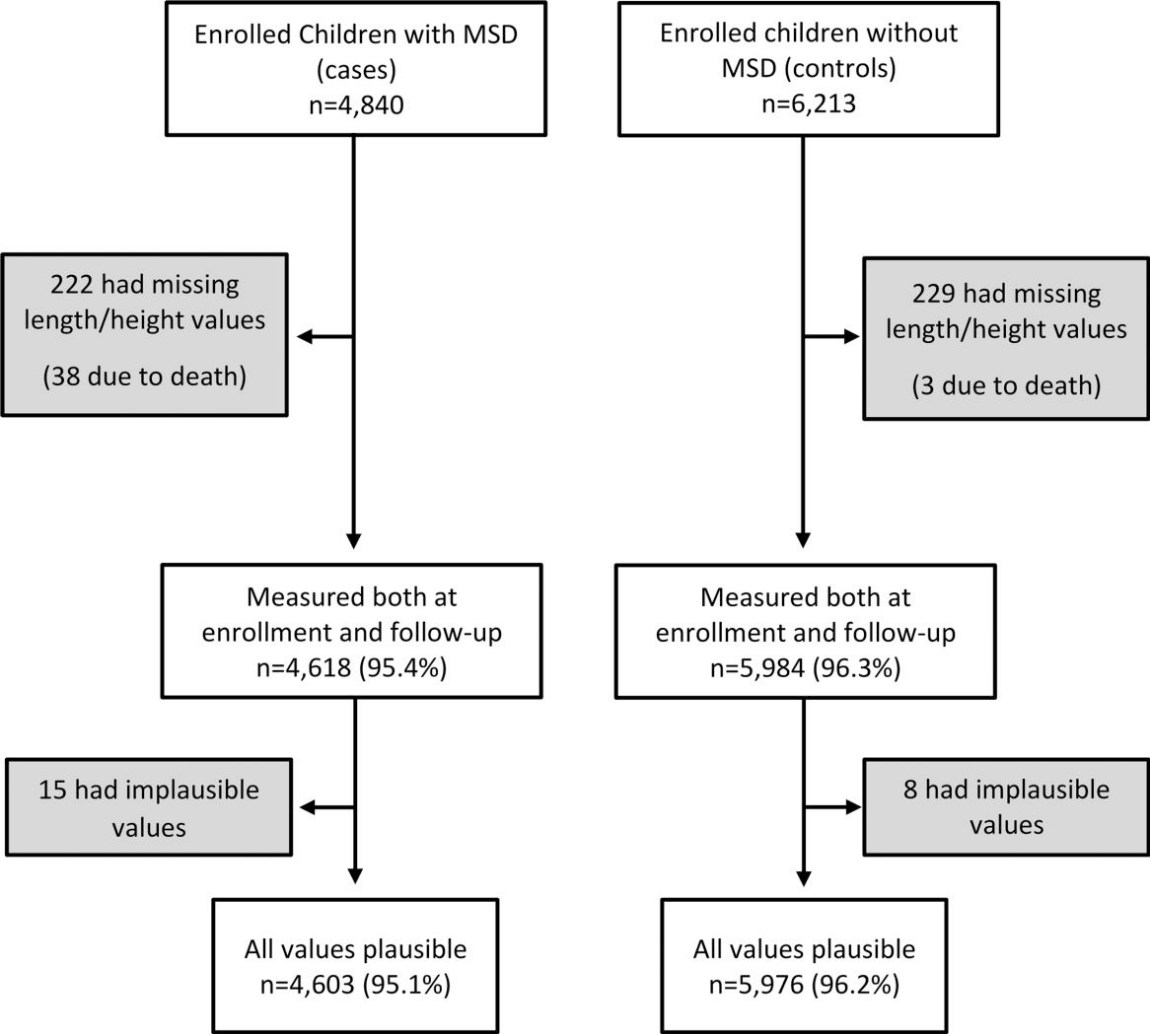
Steps of anthropometric data cleaning to derive analyzable samples. Abbreviation: MSD, moderate-to-severe diarrhea.

A total of 4840 children with MSD and 6213 matched children without MSD were enrolled. After removing missing height measurements, death, and biologically implausible measurements, 4603 (95.1%) children with MSD and 5976 (96.2%) children without MSD remained (Figure 1).

A comparison of sociodemographic factors between children with and without MSD (all sites combined) revealed several significant differences (Table 1). Compared with children without MSD, children with MSD were more likely to live in a household that had more than 2 children under the age of 5 years per caretaker (49.9% vs 46.5%; *P* < .001) and to own a scooter (51.4% vs 49.1%; *P* = .02); they were less likely to own agricultural land (57.0% vs 60.1%; *P* = .001) (Table 1). Compared with children without MSD, children with MSD were less likely to have access to improved drinking water (84.1% vs 87.2%; *P* < .0001) but more likely to have access to improved sanitation (62.8% vs 56.6%; *P* < .0001) (Table 1). The average duration of an episode of diarrhea was longer for children with MSD compared with children without MSD at enrollment who subsequently developed diarrhea according to the memory aid.

There was a trend of an increased proportion of stunted children with increasing age strata in children with MSD (14.5%, 25.1%, and 26.6%) and without MSD (13.7%, 25.6%, and 24.7% at enrollment). In The Gambia and Kenya, this was seen across all age strata, whereas in Mali, the increase tapered off in the oldest age stratum. During the first 2 years of life, this trend was reflected in the finding of a significantly higher proportion of both groups being stunted at follow-up compared with at enrollment across all study sites, with the exception of Malian children without MSD aged 12–24 months (*P* = .75) (Table 2).

**Table 2.** Prevalence of Stunting at Enrollment and Follow-up in Children With Moderate-to-Severe Diarrhea (MSD) and Children Without MSD During May 2015 to July 2018, by Site and Age

	The Gambia			Mali			Kenya		
Age Group	Children With MSD, n (%)	Children Without MSD, n (%)	*P* ^a^	Children With MSD, n (%)	Children Without MSD, n (%)	*P* ^a^	Children With MSD, n (%)	Children Without MSD, n (%)	*P* ^a^
**0–11 Months, n**	512	670		562	665		555	700	
ȃStunted at enrollment	82 (16.0)	100 (14.9)	.66	52 (9.3)	59 (8.9)	.90	102 (18.4)	119 (17.0)	.57
ȃStunted at follow-up	128 (25.0)	150 (22.4)	.33	84 (14.9)	84 (12.6)	.27	140 (25.2)	152 (21.7)	.16
ȃ*P* value^b^	<.0001*	<.0001*		<.0001*	.0006*		<.0001*	<.0001*	
**12–23 Months, n**	581	715		532	620		505	703	
ȃStunted at enrollment	161 (27.7)	215 (30.1)	.38	104 (19.5)	99 (16.0)	.13	141 (27.9)	208 (29.6)	.57
ȃStunted at follow-up	194 (33.4)	238 (33.3)	1.00	124 (23.3)	102 (16.5)	.004*	171 (33.9)	229 (32.6)	.68
ȃ*P* value^b^	<.0001*	.002*		.002*	.75		<.0001*	.004*	
**24–59 Months, n**	486	661		448	611		422	631	
ȃStunted at enrollment	169 (34.8)	207 (31.3)	.24	80 (17.9)	78 (12.8)	.03*	112 (26.5)	185 (29.3)	.36
ȃStunted at follow-up	178 (36.6)	213 (32.2)	.14	78 (17.4)	77 (12.6)	.04*	121 (28.7)	181 (28.7)	1.00
ȃ*P* value^b^	.16	.36		.83	1.00		.066	.48	

Data are presented as n (%) unless otherwise indicated. Abbreviation: MSD, moderate-to-severe diarrhea.

Chi-square test of association between stunting status (yes/no) at enrollment and MSD/diarrhea-free status, similarly for stunting status at follow-up.

McNemar's test comparing the proportion of stunting at enrollment to the proportion stunted at follow-up within each MSD/diarrhea-free group.

*Significant, *P* < .05.

When each age/site stratum was examined, similar proportions of children with and without MSD were stunted at enrollment, with the exception that Malian children 24–59 months old with MSD were more likely to be stunted than those without MSD (17.9% vs 12.8%; *P* = .03) (Table 2). Similarly, the proportion of children in both groups who were stunted at follow-up was comparable, with the exception of Malian children aged 12–23 months (23.3% vs 16.5%; *P* = .004) and 24–59 months (17.4% vs 12.6%; *P* = .04), where children without MSD experienced significantly less stunting.

Notably, among children who were not stunted at enrollment, a higher proportion of children with MSD became stunted at follow-up compared with children without MSD (8.0% vs 5.5%; *P* < .0001) (Table 3). In contrast, 92.7% of children with MSD and 91.8% children without MSD who were stunted at enrollment continued to be stunted at follow-up (*P* = .47) (Table 3).

**Table 3.** Change in Stunting (HAZ <−2) Status in Children Between Enrollment and at ∼60-Day Follow-up Visit

Predictors	Children With MSD, n (%)	Children Without MSD, n (%)	*P* ^a^
**Not stunted at enrollment, n**	3600	4706	
ȃNot stunted at follow-up	3312 (92.0%)	4446 (94.5%)	<.0001*
ȃStunted at follow-up	288 (8.0%)	260 (5.5%)	
**Stunted at enrollment, n**	1003	1270	
ȃNot stunted at follow-up	73 (7.3%)	104 (8.2%)	.47
ȃStunted at follow-up	930 (92.7%)	1166 (91.8%)	

Abbreviations: HAZ, height-for-age *z*-score; MSD, moderate-to-severe diarrhea.

*P* values from chi-square tests comparing children with and without MSD.

*Significant, *P* < .05.

The final GLMM included 4 interaction terms and the estimated effects are summarized in Table 4. Among children not stunted at enrollment, children with MSD were significantly more likely (by a factor of 30%) to be stunted at follow-up than those without MSD, after controlling for other factors (adjusted odds ratio [aOR]: 1.30; 95% confidence interval [CI]: 1.05–1.62; *P* = .018). Among children who were stunted at enrollment, children from Kenya had higher odds of stunting at follow-up compared with children from Mali (aOR: 1.90; 95% CI: 1.12–3.23; *P* = .018). In contrast, among those already stunted at enrollment, an episode of MSD was not significantly associated with the odds of being stunted at follow-up overall (aOR: 1.02; 95% CI: .72–1.44; *P* = .92). However, those aged 0–11 and 12–23 months were significantly more likely to be stunted at follow-up compared with 24–59-month-old children (aOR: 2.65 [95% CI: 2.04–3.46; *P* < .001] for 0–11 months and 2.26 [95% CI: 1.72–2.97; *P* < .001] for 12–23 months). Additionally, among children not stunted at enrollment, males had significantly higher odds of being stunted at follow-up compared with females (aOR: 1.39; 95% CI: 1.16–1.67; *P* < .001). Other variables that significantly increased the odds of stunting at follow-up included a longer duration of follow-up and caretakers with more than 2 under-5 children in their care, whereas caretakers with a primary education or higher, use of clean cooking fuel, and possession of electricity, television, and refrigerator had children with a significantly lower odds of stunting at follow-up (Table 4).

**Table 4.** Adjusted Effect of Moderate-to-Severe Diarrhea on the Odds of Stunting (HAZ <−2) at Follow-up

Predictors	aOR^a^ (95% CI)	*P*
**Not stunted at enrollment**		
ȃChildren without MSD	1	
ȃChildren with MSD	1.30 (1.05, 1.62)	.018*
ȃȃAge stratum		
ȃȃȃ24–59 months	1	
ȃȃȃ0–11 months	2.65 (2.04, 3.46)	<.001*
ȃȃȃ12–23 months	2.26 (1.72, 2.97)	<.001*
ȃȃSite		
ȃȃȃMali	1	
ȃȃȃThe Gambia	1.14 (.78, 1.68)	.50
ȃȃȃKenya	1.08 (.70, 1.64)	.74
ȃȃMale vs female	1.39 (1.16, 1.67)	<.001*
**Stunted at enrollment**		
ȃChildren without MSD	1	
ȃChildren with MSD	1.02 (.72, 1.44)	.92
ȃȃAge stratum		
ȃȃȃ24–59 months	1	
ȃȃȃ0–11 months	.86 (.56, 1.31)	.48
ȃȃȃ12–23 months	1.00 (.69, 1.45)	.99
ȃȃSite		
ȃȃȃMali	1	
ȃȃȃThe Gambia	1.36 (.84, 2.21)	.21
ȃȃȃKenya	1.90 (1.12, 3.23)	.018*
ȃȃMale vs female	.96 (.69, 1.33)	.79
More than 2 under-5 children per caretaker (vs ≤2 children/caretaker)	1.32 (1.07, 1.62)	.008*
Crowding (>3 people sleeping per room vs ≤3 people/room)	1.05 (.89, 1.23)	.55
Caretaker with at least primary school education (vs less than primary education)	.81 (.67, .99)	.037*
Household possessions		
ȃCar (vs no car)	.89 (.68, 1.19)	.44
ȃScooter (vs no scooter)	1.08 (.88, 1.32)	.45
ȃBike (vs no bike)	.95 (.79, 1.14)	.58
ȃCart (vs no cart)	.99 (.76, 1.28)	.92
ȃPhone (vs no phone)	1.09 (.71, 1.68)	.70
ȃRadio (vs no radio)	.92 (.75, 1.12)	.39
ȃAgricultural land (vs no land)	1.20 (.89, 1.62)	.22
Principal component ETR (electricity, television, refrigerator)	.92 (.85, .99)	.027*
Household with the finished floor (vs unfinished floor)	.93 (.73, 1.17)	.52
Household uses clean cooking fuels (vs unclean fuels)	.61 (.39, .96)	.031*
Improved drinking Water source (vs unimproved source)	.83 (.66, 1.04)	.10
Access to improved sanitation (vs unimproved sanitation)	.94 (.77, 1.14)	.52
History of pneumonia before ∼60 days follow-up	1.12 (.62, 2.03)	.71
Days to follow-up	1.03 (1.02, 1.04)	<.001*
Duration of diarrhea, days	1.02 (.99, 1.04)	.15

Abbreviations: aOR, adjusted odds ratio; CI, confidence interval; HAZ, height-for-age *z*-score; MSD, moderate-to-severe diarrhea.

Adjusted odds ratio based on the generalized linear mixed-effects model with all predictors listed in this table and the interaction between MSD and enrollment stunting status (*P* = .19), the interaction between age strata and enrollment stunting status (*P* < .001), the interaction between site and enrollment stunting status (*P* = .051), and the interaction between sex and enrollment stunting status (*P* = .052).

*Significant, *P* = .05.

## DISCUSSION

We presented updated data from sub-Saharan Africa on the risk of stunting following MSD after rotavirus vaccine introduction and high rates of coverage [14]. An episode of MSD was associated with 30% higher odds of stunting during the 50–90 days after the episode among children who were not stunted at baseline, after controlling for potential socioeconomic and other confounders.

Numerous studies have found a strong association between diarrhea and stunting among children living in low-resource settings [22, 23]. Richard and colleagues [24] analyzed anthropometry and diarrheal surveillance data among children under 2 years of age from 7 cohort studies conducted over a period of 2 decades in 4 countries, including Peru, Brazil, Guinea-Bissau, and Bangladesh, and demonstrated an association between diarrhea prevalence (days with diarrhea) and linear growth faltering. Catch-up growth was observed that potentially allowed children to regain their original trajectories but was predicated on the child experiencing diarrhea-free periods. In many low-resource settings where children are experiencing frequent episodes of diarrhea, they do not have enough diarrhea-free periods to catch up on growth. Recurrent infections during childhood along with nutritional deficiencies may result in long-term linear growth faltering in these children. Children may be subject to excessive mortality immediately after an episode, as has been observed during the 2–3-month period of an episode of MSD [11]. It is notable that a follow-on study to GEMS, designated GEMS-1A, demonstrated that children with less severe diarrhea (medically attended diarrhea not meeting criteria for MSD) had comparable levels of linear growth faltering to those with MSD, substantially increasing the number of young children who experience diarrhea-related linear growth sequelae [25]. Research is needed to explore interventions that might be effective for the prevention of diarrhea and the extension of diarrhea-free periods in early childhood. Protein-rich nutritional supplementation was found to either decrease growth faltering or increase the chances of catch-up linear growth after diarrhea in Colombia [26], suggesting that these and other promising nutritional interventions merit further investigation [27].

Results from our study show that the age at MSD presentation is an important predictor of stunting among children not previously stunted, with the highest risk of stunting in the first 2 years of life, as reported elsewhere [11], confirming the need to focus on children during the first 1000 days [28, 29]. Male children had higher odds of post-MSD stunting compared with females, which is consistent with other studies [24, 30, 31]. This sex-based inequality has been attributed, at least in part, to a higher burden of diarrhea and other illnesses and a greater zinc deficiency in boys, which is associated with growth faltering and increased incidence and severity of diarrhea [24]. These results also point to the need to account for context- and site-specific differences. In this study, we observed that children who were stunted at enrollment from Kenya had higher odds of stunting compared with those who were stunted at enrollment from Mali.

We found that asset ownership and maternal education were associated with less stunting at follow-up. Maternal education, in particular, a proxy for improved childcare practices related to health and nutrition, has been associated with decreased stunting in children overall [30–34]. A higher asset index predicted substantial improvements in HAZ in several countries and may reflect similar behaviors [34, 35].

Several limitations need to be considered in interpreting our findings. Most important, the lack of reliable data on key variables such as birth length and infant-feeding practices may influence the relationship between MSD and stunting and needs to be considered. Although a previous study has shown that breastfeeding practices did not confound the effect of diarrhea on linear growth [23], we were unable to evaluate the effects of the duration of breastfeeding, which has been associated with stunting in some studies [36]. It is possible that caretakers change their feeding practices, especially when a child is sick, which may contribute to differences in nutrient intake and protection from infection among children with MSD compared with those without MSD. Although we asked about other infections and care-seeking during the 2–3 months following MSD, the results relied on caregiver recall and we also lacked reliable information on treatment of subsequent illnesses following an episode of MSD. Future studies should include additional information on dietary intakes, access to health care, coinfections, seasonality, diarrhea etiologies, and intestinal inflammation. Studies with a longer period of follow-up can increase understanding of the impact of multiple episodes of diarrhea on stunting. It is also important to explore whether individual pathogens differentially impact linear growth. The strengths of this study include a prospective design with a large sample size from 3 sites and use of standardized procedures.

In summary, an episode of MSD during the first 5 years of life was associated with a higher odds of stunting within 2–3 months after the episode. Diarrhea, especially MSD, is preventable in early childhood, and efforts to address this important risk should be fully integrated into programs aimed at reducing childhood stunting.

## Notes

***Acknowledgments.*** The authors express their deep gratitude to the families who participated in these studies, the clinical and field staff for their exceptional hard work and dedication, and to the physicians, administration, and health officials at every site who generously provided facilities and support for the conduct of the study. They are grateful to Catherine Johnson, Chris Focht, and Nora Watson at the Emmes Company, LLC, for expert data management and reporting. Special thanks go to Carl Kirkwood, Duncan Steele, and Anita Zaidi at the Bill & Melinda Gates Foundation for helpful oversight; Kathy Neuzil for thoughtful suggestions; and to the following members of our International Scientific Advisory Committee for providing insightful comments and guidance: Janet Wittes (Chair), George Armah, John Clemens, Christopher Duggan, Stephane Helleringer, Ali Mokdad, James Nataro, and Halvor Sommerfelt.

***Financial support.*** This work was supported by the Bill & Melinda Gates Foundation grants OPP1111236 and OPP1116751 (to K. M. Neuzil).

***Supplement sponsorship.*** This supplement was sponsored by the Bill & Melinda Gates Foundation. This study is based on research funded in part by grants from the Bill & Melinda Gates Foundation (OPP1111236/OPP1116751).
